# Mental health status of infertile couples based on treatment outcome

**Published:** 2013-06

**Authors:** Mohammad Hosein Baghianimoghadam, Amir Hossein Aminian, Behnam Baghianimoghadam, Nasrin Ghasemi, Ali Mohammad Abdoli, Najmeh Seighal Ardakani, Hosein Fallahzadeh

**Affiliations:** 1 *Department of Health Services, Faculty of Health, Shahid Sadoughi University of Medical Sciences, Yazd, Iran.*; 2 *Department of Clinical Psychology, Shahid Sadoughi University of Medical Sciences, Yazd, Iran.*; 3 *Research and Clinical Center for Infertility, Shahid Sadoughi University of Medical Sciences, Yazd, Iran.*; 4 *Faculty of Health, Shahid Sadoughi University of Medical Sciences, Yazd, Iran.*; 5 *Department of Biostatistics, Faculty of Health, Shahid Sadoughi University of Medical Sciences, Yazd, Iran.*

**Keywords:** *Infertility*, *General health questionnaire*, *Mental health*, *Treatment outcome*

## Abstract

**Background:** Infertility is accompanied by numerous psychological and social problems. Infertile couples are more anxious and emotionally distressed than other fertile people. Previous studies suggested that infertility is more stressful for women than men.

**Objective: **The purpose of this study was to determine the status of general health of infertile couples.

**Materials and Methods:** This cross-sectional study evaluated general health of 150 infertile couples attending to Yazd Research and Clinical Center for Infertility that were selected consequently. The data were gathered by the researchers, based on face to face interview before and after three months of treatment by two questionnaires. The first questionnaire had questions on demographic information and the second one was the General Health Questionnaire-28 (GHQ-28). This questionnaire has four sub- scales areas. All data were transferred directly to SPSS 15 and analyzed.

**Results: **The mean age of women was 28.3 and men were 32.4 years. The scores for all sub- scales of GHQ in women were more than men. There was significant difference between age and general health at physical symptoms scales (p=0.002), anxiety and sleep disorders (p=0.003). The age group 25-29 years had higher scores (more than 7) than other age groups. There was significant difference between the scale of social dysfunction and results of treatment.

**Conclusion:** Our results, similar to the previous studies have revealed negetive social and mental effects of infertility on women is more than men, so there is need that they be educated specially.

## Introduction

Although infertility as a psychological stressor can threat health of infertile people; but the extent of its effects is depended to cognitive and defense skills of people (1-3). Infertility is a major tension in life, imposes much stress on couples and can threat psychological health of them by effect on quality of married life, fear of possible divorce, decreasing intimacy, despair and depression or other psychological disorders like anxiety, irritability, lack of self-efficacy, sexual disorders etc. (4).

Treatment approaches for infertile couples are developing; treatment with new technologies has much stresses. Also the effect of these stresses can impact on treatment success (5). Evaluation of quality of life and effects of psychological treatment interventions (counseling or drugs) in infertile people can make an efficient step on infertility treatment success (6).

Psychological effects of infertility on life of infertile couple are concomitant with tensions between families and also unsatisfaction of their marriage (7). Due to increased global population and higher age of marriage, number of infertile couples is increasing. Prevalence of infertility is different in different countries. Some data shows that there are about 80 million infertile couples worldwide. In Iran estimations indicate that there are about two million infertile couples (8). 

World Health Organization (WHO) indicates that infertility is a main problem in fertility health that has different physical, psychological and social dimensions (9). Psychological pressures and concerns about infertility have direct effects on normal physiology of body and can have double effect on fertility outcomes (10). People that have peace of mind and are psychologically healthy, are less under stresses and then have more probability of successful fertility outcomes (11). 

Regardless of the technique which the couple use for treatment of infertility (like IVF, ART, surrogacy and adoption), successful outcome can improve quality of life and general health (12). Progression of the infertility treatment modalities had opened most of locks in the way of infertility problems; but had some own complications too. Various studies showed that developed treatment methods such as IVF can cause depression and anxiety symptoms among 5-10% of women (13, 14).

Experience of pregnancy, delivery and giving love to the own child, is a natural need in woman. Typically women know their first role as being a mother and infertility can cause the sense of inability and incompetence. On the other hand, infertile women are more involved in long time strict medications and the high cost of infertility treatments that can have psycho-social reaction in infertile women (15). If physician give the assurance that a couple will not be able to have child, this may cause psychological crisis that can effect on their communications and job skills (16). Some researchers believe that a complicated intertwined network of physical, psychological and social factors can affect infertile individuals and the reaction of patient to these situations can form the pattern of patient response to the problem (17). 

The aim of our study is to determine the situation of general failure of infertile women after successful and unsuccessful treatment, to design appropriate educational programs and lower the difficulties of patients.

## Materials and methods

This cross-sectional descriptive-analytic study was done during March 2009 to February 2010 among infertile couples who admitted in Research and Clinical Center for Infertility, Yazd, Iran. This study was approved by Shahid Sadoughi University of Medical Sciences Ethics Committee. With considering 95% confidence interval and study power of 80% and d=0.15 (according to the proportion of general health between two groups), 140 patients were needed for the study. Patients were chosen consequently with respect to the day which they attended to the center. 

Patients with previous history of infertility treatment were excluded from the study. Questionnaires were completed with interviewing by a trained public health Master of Science graduate till the needed cases were reached. The aim of study was explained to each participant and after taking consent they included in the study. Data were collected by two questionnaires. One was made by researcher about the demographic data and the second one was GHQ-28 (General Health Questionnaire-28). GHQ is the most known questionnaire for screening in psychology that has been designed by Goldberg and Hilir (18). 

This questionnaire encompasses two major phenomenons, one inability to continue normal function and the second presence of the phenomenon with distressing nature. This questionnaire can detect discomforts with duration lower than four weeks and can be used for studies on detecting background precipitating factors of general population or specific groups; and also comparison of psychological syndromes in a population (19). This questionnaire is composed of 28 questions which are divided in four parts with seven questions in each part. 

The first part is about physical symptoms (like self-assessment of patient on his health or the sense of fatigue). The second part covers symptoms of anxiety like sense of pressure, confusion, angry, fear without cause or inability of doing things. The third part is about social dysfunction, the questions are on ability of subject to do his or her ordinary daily works, satisfaction in doing tasks, feeling of being useful, ability to learn and enjoying daily activities. 

The fourth part asks about the symptoms of major depression like anhedonia, hopelessness, suicidal thoughts, dying wish and inability in doing tasks. Scoring is based on four-way classification that subject give scores of 0-3 to each question. Iranian studies shows that the cut point on score 23 is the determinant extent border for this questionnaire in Iranian population. Then scores between 0-23 will be considered as appropriate general health and more than 24 will be considered as inappropriate general health. Validity of this questionnaire has been confirmed by health professionals and also has been used in several previous studies in Iran (20-23).

Questionnaires were completed firstly at the time of admission in Research and Clinical Center for Infertility of Yazd. Patients were undergone treatment. After three months of treatment the questionnaire was completed at the second stage of study. Variables including physical symptoms scales, anxiety and sleep disorders scale, social function scale and depressive symptoms scales were evaluated and analyzed. We used cronbach’s alpha test for internal consistency that was calculated 0.93 for all study questions. 

**Statistical analysis**

All registered data were transformed to SPSS 15, and analyzed under Mann-Whitney and Kruskal-Wallis tests. Results with p<0.05 were statistically significant.

## Results

Totally 150 infertile couples (150 men and 150 women) were included in the study. Nineteen couples (totally 38 participants) did not participated in the second stage of the study and were excluded from the study. Twenty two participants were illiterates, 55 finished primary school, 131 had high school diploma and 92 had academic degrees. 71 couples had family relationships and 79 had not. Age of 37 subjects was 20-24 years, 106 were 25-29 years old, 89 were 30-34 years old and 68 were more than 34 years old. 

Cause of infertility for 47 couples was female factor, 44 was male factor, 16 was both female and male factor and for 42 couples was undetermined. Also 250 of participant had no family history of infertility but 50 subjects had such a history. Participants who discontinued the treatment had higher GHQ scores at all scales contrasting with people who had successful or even unsuccessful treatment outcomes, except anxiety and sleep disorders symptoms that patients with unsuccessful treatment outcomes had worse scores. 

There was no significant relation between social function and treatment outcome but other variables had significant discrepancy with treatment outcome (Table I). The scores of anxiety symptoms and sleep disorders for subjects who had successful treatment comparing with subjects who gave up treatment or had unsuccessful outcomes were 5.57 contrasting with 7.46 and 7.09 at women and 3 contrasting with 4.55 and 5.08 in men respectively.

There was only relation between level of graduation and physical symptoms scales before and after treatment (p=0.046). But generally scores of patients with lower literature was higher. Social function score in this group was 8.5 that will be in the range of at risk group. Couples with low economic situation had significantly higher scores according to general health in two scales of physical symptoms scales (p=0.004) and depression symptoms (p=0.001) before and after treatment. In depression symptoms, none of groups were not as at risk population (score more than 7), but in other scales relatively all groups were in at risk range (Table II).

The scores of couple’s with the cause of infertility both male and female factor, were higher than other groups (more than 7) and were in range of at risk population. There was significant relation between familial relationship of couples and all scales of GHQ (Table III). There was significant difference between age and general health at physical symptoms scales, anxiety and sleep disorders (p=0.002 and p=0.003 respectively) that age group 25-29 years had higher scores (more than 7) than other age groups (Table IV). Also there was significant relationship between sex and general health as women had higher scores at all scales (Table V). Also we found no significant relationship between duration of marriage and scales of GHQ.

**Table I:** Mental health status of the study participants based on treatment outcome

** Scales**	**Physical symptoms scales (Mean ± SD)**	**Anxiety and sleep disorders scale (Mean ± SD)**	**Social function scale (Mean ± SD)**	**Depressive symptoms scales (Mean ± SD)**
**Treatment status**				
Discontinue treatment	54 (5.82 ± 4.42)	6.03 ± 4.58	7.69 ± 3.81	2.18 ± 4.13
Negative treatment results	152 (5.14 ± 2.69)	6.1 ± 4.55	7.59 ± 3.64	2.86 ± 3.8
Got pregnant	56 (3.26 ± 2.59)	4.28 ± 2.52	6.52 ± 3.45	1.17 ± 1.95
Total	262 (4.88 ± 2.96)	5.7 ± 4.41	7.38 ± 3.65	2.53 ± 3.64
P-value	0.001	0.011	0.075	0.001

Mann whitney test

**Table II:** Mental health status of the study participants based on economic status before and after treatment

		**Physical symptoms scales** **(Mean ± SD)**		**Anxiety and sleep disorders scale ** **(Mean ± SD)**		**Social function scale** **(Mean ± SD)**		**Depressive symptoms scales ** **(Mean ± SD)**	
	**N**	**Before**	**After**	**Before**	**After**	**Before**	**After**	**Before**	**After**
Prosperous	6	4 ± 2	3 ± 2	4.8 ± 1.09	4 ± 2.64	7.8 ± 1.79	8.66 ± 2.08	0.2 ± 0.45	0.33 ± 0.57
Relatively prosperous	12	4.14 ± 3.9	3.35 ± 2.67	4.71 ± 3.97	5.5 ± 4.01	2.93 ± 3.41	6.42 ± 2.92	1.64 ± 2.1	1.71 ± 2.64
The average income	192	4.66 ± 3.3	4.56 ± 3.74	5.39 ± 4.25	5.28 ± 4.25	6.81 ± 2.38	7.25 ± 3.52	2.27 ± 3.35	2.1 ± 3.16
Low income	38	6.37 ± 4.55	6.78 ± 4.93	6.6 ± 5.13	7.58 ± 5.05	6.16 ± 2.8	8.24 ± 4.22	3.48 ± 4.6	4.36 ± 4.81
Poor	14	6.63 ± 2.9	5.69 ± 3.32	7.8 ± 4.6	6.53 ± 3.95	7.42 ± 3.93	7.3 ± 4.42	6.37 ± 5.06	4.38 ± 5.04
Total	262	5.03 ± 3.59	4.88 ± 3.96	5.7 ± 4.42	5.7 ± 4.4	6.85 ± 2.61	7.38 ± 3.65	2.66 ± 3.78	2.53 ± 3.64
P-value		0.004		0.138		0.537		0.001	

Kruskal-Wallis

**Table III:** Mental health status of the study participants based on familial relationship before and after treatment

** Scales**		**physical symptoms scales** **(Mean ± SD)**		**Anxiety and sleep disorders scale (Mean ± SD)**		**Social function scale** **(Mean ± SD)**		**Depressive symptoms scales (Mean ± SD)**	
**Family relations**									
	**N**	**Before**	**After**	**Before**	**After**	**Before**	**After**	**Before**	**After**
No	220	4.87 ± 3.31	4.82 ± 3.9	5.4 ± 4.12	5.59 ± 4.35	6.77 ± 2.43	7.33 ± 3.59	2.43 ± 3.48	2.4 ± 3.43
Yes	42	5.8 ± 4.71	5.21 ± 4.30	7.18 ± 5.46	6.28 ± 4.68	7.22 ± 3.22	7.64 ± 3.99	3.82 ± 4.92	3.19 ± 4.57
Total	262	5.03 ± 3.59	4.88 ± 3.96	5.7 ± 4.42	5.7 ± 4.4	6.85 ± 2.61	7.38 ± 3.65	2.66 ± 3.78	2.53 ± 3.64
P-value		0.021		0.017		0.001		0.003	

Mann whitney test

**Table IV:** Health status of the study participants based on age of them before and after treatment

**Age (years)**	**N**	**physical symptoms scales** **(Mean ± SD)**		**Anxiety and sleep disorders scale ** **(Mean ± SD)**		**Social function scale** **(Mean ± SD)**		**Depressive symptoms scales** **(Mean ± SD)**	
		**Before**	**After**	**Before**	**After**	**Before**	**After**	**Before**	**After**	
20-24	34	5.95 ± 3.61	4.73 ± 2.97	5.43 ± 4.91	4.86 ± 4.01	7.05 ± 2.3	8 ± 3.83	3.3 ± 4.38	2.56 ± 3.33	
25-29	92	5.73 ± 3.86	6.1 ± 4.67	6.8 ± 4.24	6.93 ± 4.68	7.18 ± 2.9	8.04 ± 3.8	3.12 ± 4.02	3.44 ± 4.15	
30-34	78	4.34 ± 3.15	4.18 ± 3.31	5.04 ± 4.36	5.24 ± 4.11	6.62 ± 2.17	6.81 ± 3.75	2.18 ± 3.54	2.4 ± 3.91	
>35	60	4.37 ± 3.44	4.1 ± 3.67	5.03 ± 4.26	4.98 ± 4.24	6.53 ± 2.84	6.84 ± 3.09	2.26 ± 3.29	1.5 ± 2.21	
Total	262	5.03 ± 3.59	4.88 ± 3.96	5.7 ± 4.42	5.7 ± 4.4	6.85 ± 2.61	7.38 ± 3.65	2.66 ± 3.78	2.53 ± 3.64	
P-value		0.002		0.003		0.142		0.071		

Kruskal-Wallis

**Table V:** Mental health status of the study participants based on sex before and after treatment

		**Physical symptoms scales** **(Mean ± SD)**		**Anxiety and sleep disorders scale (Mean ± SD)**		**Social function scale** **(Mean ± SD)**		**Depressive symptoms scales ** **(Mean ± SD)**	
	**N**	**Before**	**After**	**Before**	**After**	**Before**	**After**	**Before**	**After**
Men	131	4.17 ± 3.02	3.33 ± 2.62	4.95 ± 3.73	4.51 ± 3.57	6.31 ± 2.49	6.44 ± 3.32	2.07 ± 3.39	1.64 ± 2.68
Women	131	5.88 ± 3.9	6.39 ± 4.44	6.45 ± 4.92	6.84 ± 4.82	7.39 ± 2.64	8.28 ± 3.73	3.25 ± 4.06	3.39 ± 4.21
Total	262	5.03 ± 3.59	4.88 ± 3.96	5.7 ± 4.42	5.7 ± 4.4	6.85 ± 2.61	7.38 ± 3.65	2.66 ± 3.78	2.53 ± 3.64
p-value		0.001		0.019		0.001		0.001	

Mann whitney test

## Discussion

Infertility has several effects on different dimensions of infertile couples. Results of our study show that the effect of infertility treatment is not similar in all persons. Diagnosis, follow up and treatment of infertility cause stress and can lead to anxiety, depression and other psychological and physical illnesses and also cause social abnormalities. 

Our study showed that mean scores for social function was higher than other scales (physical, anxiety symptoms, sleep disorders and depression symptoms scales). The lowest scores were for depression symptoms. These results are concomitant with Shaker *et al* study, which showed that 42% of subjects had social dysfunction but only 6% had depression (24). High prevalence of mental disorders at under treatment infertile women is related to cultural factors too. Several studies punctuate on the role of being mother as the most important factor that satisfies the sense of womanhood. Then women, who have problem to do this role, will be exposed to more sever stresses. 

Infertility and stresses of treatment can cause somatization disorders and social dysfunction (25). Sometime symptoms of disease are more prevalent than the disease itself. Depression is a disorder that can be presented with sleep disorders and anxiety. The remarkable point in our study was the low score of depression symptoms in our study while in most of other studies depression was one of the most prevalent problems among infertile women (26). 

These results could be due to texture of our participants. Based on Table II there was significant relationship between income and depressive symptoms as scores for high and middle income groups is apparently higher than lower income groups, and in this study most of our participants had middle income; people who mostly live in urban areas and have higher education levels. They have better concept on the disease its treatment and also have more financial capabilities which lower concerns about the possible needed repeated expensive treatment courses. High level of stress, fear of loneliness and treatment failure cause anxiety and somatic symptoms in infertile women. 

Failure to cope with the stressful situation hampers patients to reasonably thinking and problem solving coping strategy. Our result show that with increasing psychological disorders symptom, the extent of emotional coping strategies will increase too. Infertile people use emotional coping strategies more due to lack of control on life events, low self-esteem, low social support and high level of stresses (27). Other studies showed that when an event have high level of threat, person evaluate it as more important issue, then attention will be focused on the emotions instead of the problem itself, then the person use emotional coping strategy more (26).

The scores of all questionnaire scales in subjects who had successful treatment outcomes were higher than subject who did not get pregnant. These results are concomitant with most of previous studies like Ghorbani *et al* study. Emotional turmoil is prevalent in people who are unsuccessful in treatment and need professional consultation (28). Scores for all parts of general health questionnaire were higher in women than men, especially after treatment in most of scales, women had scores more than 7 that was higher than men and made them as at risk population. 

Scores of women according to depression symptoms at all three groups (patients who discontinue treatment, were unsuccessful in treatment or were successful) was lower than other scales symptoms. Social dysfunction scores was higher than 7 at all three mentioned groups. Men also had high social dysfunction scores but was not more than 7 at any group. Sadeghian *et al* concluded that depression, fear, anxiety, somatization disorders and feeling of hostility at infertile women are more than infertile men (29). 

Based on our results infertility is effective on anxiety in both men and women and successful treatment can lower the level of anxiety. The scores of anxiety symptoms and sleep disorders for subjects who had successful treatment comparing with subjects who gave up treatment or had unsuccessful outcomes was 5.57 contrasting with 7.46 and 7.09 at women and 3 contrasting with 4.55 and 5.08 in men respectively. These results in our study don’t confirm some other similar studies which concluded that anxiety of infertile women had no effect on treatment outcome (30, 31). 

Our study and also Behdani *et al* study concluded that at all scales of general health, except social function, the scores were higher in illiterate or low graduated subjects than patients with high school diploma or academic graduations (32). Probably couples with higher graduations, because of better knowledge on infertility, better coping to problem, more appropriated adaptive mechanism and support from spouse, are less likely to depression symptoms development.

In our study scores of general health scales were lower with increasing age, then the situation of general health was better in high ages. The differences were significant for anxiety and sleep disorders scores. In a study was done in turkey results shows that with increasing the age and the duration of infertility, the severity of depression will decrease (33). This may be due to coping with the problem because of long time infertility. 

Our study and some other studies indicated that the mental disorders are more serious in women who are under treatment of IVF (In Vitro Fertalization) (34). There was no relation between duration of marriage and general health scales. The lowest scores were for women who were married for 5-9 years. About anxiety and sleep disorders scales, the best scores were for women who were married for 10-14 years. About depression, the lowest symptoms were seen in women who were married lower than 5 years, but differences were not significant at any scale. Our result doesn't confirm results of Abedinia *et al* that found statistical significant relationship between duration of marriage and depression and anxiety symptoms (35).

## Conclusion

Based on our study, infertile couples are at risk of psychosocial problems and it is needed to be under psychological consultations parallel with their treatment. This is more critical in women and at 24-29 years of age. Also they should be educated about their disease and process of treatment to lower the stress. 
